# Carbonic anhydrase IX-related tumoral hypoxia predicts worse prognosis in breast cancer: A systematic review and meta-analysis

**DOI:** 10.3389/fmed.2023.1087270

**Published:** 2023-03-17

**Authors:** Warapan Numprasit, Supaporn Yangngam, Jaturawitt Prasopsiri, Jean A. Quinn, Joanne Edwards, Chanitra Thuwajit

**Affiliations:** ^1^Division of Head Neck and Breast Surgery, Department of Surgery, Faculty of Medicine Siriraj Hospital, Mahidol University, Bangkok, Thailand; ^2^School of Cancer Sciences, Wolfson Wohl Cancer Research Centre, University of Glasgow, Glasgow, United Kingdom; ^3^Department of Immunology, Faculty of Medicine Siriraj Hospital, Mahidol University, Bangkok, Thailand

**Keywords:** breast cancer, carbonic anhydrase IX, meta-analysis, prognosis, survival

## Abstract

**Background:**

Tumoral hypoxia is associated with aggressiveness in many cancers including breast cancer. However, measuring hypoxia is complicated. Carbonic anhydrase IX (CAIX) is a reliable endogenous marker of hypoxia under the control of the master regulator hypoxia-inducible factor-1α (HIF-1α). The expression of CAIX is associated with poor prognosis in many solid malignancies; however, its role in breast cancer remains controversial.

**Methods:**

The present study performed a meta-analysis to evaluate the correlation between CAIX expression and disease-free survival (DFS) and overall survival (OS) in breast cancer.

**Results:**

A total of 2,120 publications from EMBASE, PubMed, Cochrane, and Scopus were screened. Of these 2,120 publications, 272 full texts were reviewed, and 27 articles were included in the meta-analysis. High CAIX was significantly associated with poor DFS (HR = 1.70, 95% CI = 1.39–2.07, *p* < 0.00001) and OS (HR = 2.02, 95% CI 1.40–2.91, *p* = 0.0002) in patients with breast cancer. When stratified by subtype, the high CAIX group was clearly associated with shorter DFS (HR = 2.09, 95% CI =1.11–3.92, *p* = 0.02) and OS (HR = 2.50, 95% CI =1.53–4.07, *p* = 0.0002) in TNBC and shorter DFS in ER^+^ breast cancer (HR = 1.81 95% CI =1.38–2.36, *p* < 0.0001).

**Conclusion:**

High CAIX expression is a negative prognostic marker of breast cancer regardless of the subtypes.

## Introduction

The incidence of breast cancer has increased in recent decades, with an estimated 13% of women developing breast cancer in their lifetime and over 40,000 deaths per year (1, 2). The survival depends on clinicopathological factors, such as tumor size, nodal status, evidence of distant metastasis as well as biological markers, including estrogen receptor (ER), progesterone receptor (PR), and human epidermal growth factor receptor 2 (HER2) status (3–5). The intrinsic breast cancer subtypes are currently significant prognostic and predictive markers. Five-year overall survival (OS) was the highest in the ER/PR-positive subtype (94%) as compared to the HER2-positive subtype (85%) and the triple-negative (TNBC) subtype (77%) (1). Breast cancer has distinct phenotypes as evidenced by patients who have a similar staging and molecular classification but have a different treatment response and prognosis (6–8). Thus, additional predictive and prognostic markers are warranted to improve the treatment and prognostic outcomes.

Tumoral hypoxia is a common characteristic of many solid tumors (9, 10). In breast cancer, median oxygen partial pressure is approximately 10 mmHg, which is less than that of the normal breast tissue (52–65 mmHg) (11, 12). Cancer cells adapt to survive under hypoxic conditions *via* hypoxia-inducible factor-1α (HIF-1α), leading to the transcription of targeted genes resulting in tumor progression and invasion (13). Subsequently, HIF-1α can trigger the transcription of targeted genes, leading to tumor progression and invasion (14).

The expression of carbonic anhydrase IX (CAIX) is targeted by the HIF-1α transcriptional activity and controls the pH between intracellular and extracellular compartments (15). It is mainly dependent on HIF-1α regulation; therefore, it can also be a marker of tumor hypoxia (16, 17). However, hypoxia is not an obligated factor, and the inactivation of the von Hippel–Lindau (VHL) gene can stabilize HIF-1α under a non-hypoxic condition and subsequently activated the CAIX overexpression (15, 18). CAIX catalyzes extracellular hydrating CO_2_ into HCO^−^_3_ and H^+^ and cooperates with other acid/base transporters to maintain extracellular acidosis and intracellular neutral/slight alkalosis (19). In contrast, CAIX-bound Cl^−^/HCO^−^_3_ exchangers (AEs) can import or provide or export HCO^−^_3_ from intracellular compartment during cell migration (20). CAIX expression mediates cancer cell growth, migration, and invasion (18) by directly binding to β-catenin, resulting in the disruption of the E-cadherin/cytoskeleton/β-catenin complex; and an acidic extracellular pH also suppresses the function of cytotoxic T-cells (21).

Many studies have shown that high CAIX expression was associated with adverse survival outcomes. In breast cancer, some studies evaluated the importance of CAIX expression in relation to survival; however, those results were controversial and mostly included a small number of patients. Ong et al. reported that CAIX expression was the independent prognostic factor for disease-free survival (DFS) and OS in TNBC. Similarly, Brennan et al. reported that high CAIX was associated with shorter OS, breast cancer-specific survival (BCSS), and relapse-free survival (RFS) (22, 23). In contrast, Currie et al. found no association between the level of CAIX and DFS and OS (24), while Pinheiro et al. reported that only a high CAIX expression was related to DFS but not to OS (25).

To address this issue, a meta-analysis was conducted to evaluate the prognostic value of CAIX in breast cancer and to determine the correlation between CAIX and breast cancer subtypes. To date, this is the first meta-analysis to focus on the prognostic role of CAIX in breast cancer. The meta-analysis revealed that a high CAIX protein expression was associated with unfavorable survival outcomes and could discriminate the prognosis in the ER-positive and TNBC subtypes.

## Materials and methods

### Search strategy

This study used EMBASE, PubMed, Cochrane, and Scopus electronic databases to search for articles. The keywords including [(Prognos*) OR (surviv*) OR (hazard) OR (disease-free) OR (“disease free”) OR (progression-free) OR (“progression-free”) OR (Kaplan–Meier) OR (“Kaplan Meier”) OR (predict*) OR (outcome) OR (efficacy) OR (effective*)] AND [(CAIX) OR (ca9) OR (“carbonic anhydrase IX”) OR (“carbonic anhydrase 9”) OR (“carbonic anhydrase-IX”) OR (“carbonic anhydrase-9”) OR (CA-IX) OR (ca-9) OR (G250)] AND [(breast cancer) OR (breast tumors*) OR (breast carcinoma)] were used.

### Selection criteria

The inclusion criteria of the present study were as follows: (a) the patients in the study cohorts who were confirmed to have invasive breast cancer, regardless of the subtype, (b) CAIX expression which was detected by immunohistochemistry (IHC), (c) the studies that reported DFS or OS with hazards ratios (HRs) and 95% confidence intervals (CIs) or the Kaplan–Meier survival curves from which HRs and 95% CIs could be extracted, and (d) the studies that were published in English. The exclusion criteria for the present study were studies that failed to meet any of the inclusion criteria, were related to non-human studies, or contained duplicated and unavailable full texts.

### Data extraction and quality assessment

The search with regard to data extraction and quality assessment was reviewed by three independent reviewers (WN, JP, and SY). The following information was extracted from each study: the first author’s name, year of publication, the total number of patients, the scoring method and cut-off level for high or low CAIX expression, breast cancer subtypes, HRs, 95% CIs of DFS and OS, and whether univariate or multivariate analysis was performed.

### Statistical methods

Pooled HRs and their 95% CIs were used to determine the association between CAIX expression and survival. Heterogeneity among studies was assessed using the chi-squared test and I^2^. A *p*-values of <0.1 or an I^2^ statistic of >50% was indicative of significant heterogeneity between studies; in these cases, a random-effects model was used. The meta-analysis was performed with Review Manager 5.4 (RevMan the Cochrane Collaboration; Oxford, England). The *p*-values of <0.05 were considered statistically significant.

## Results

### Study selection and characteristics

**Figure 1:**
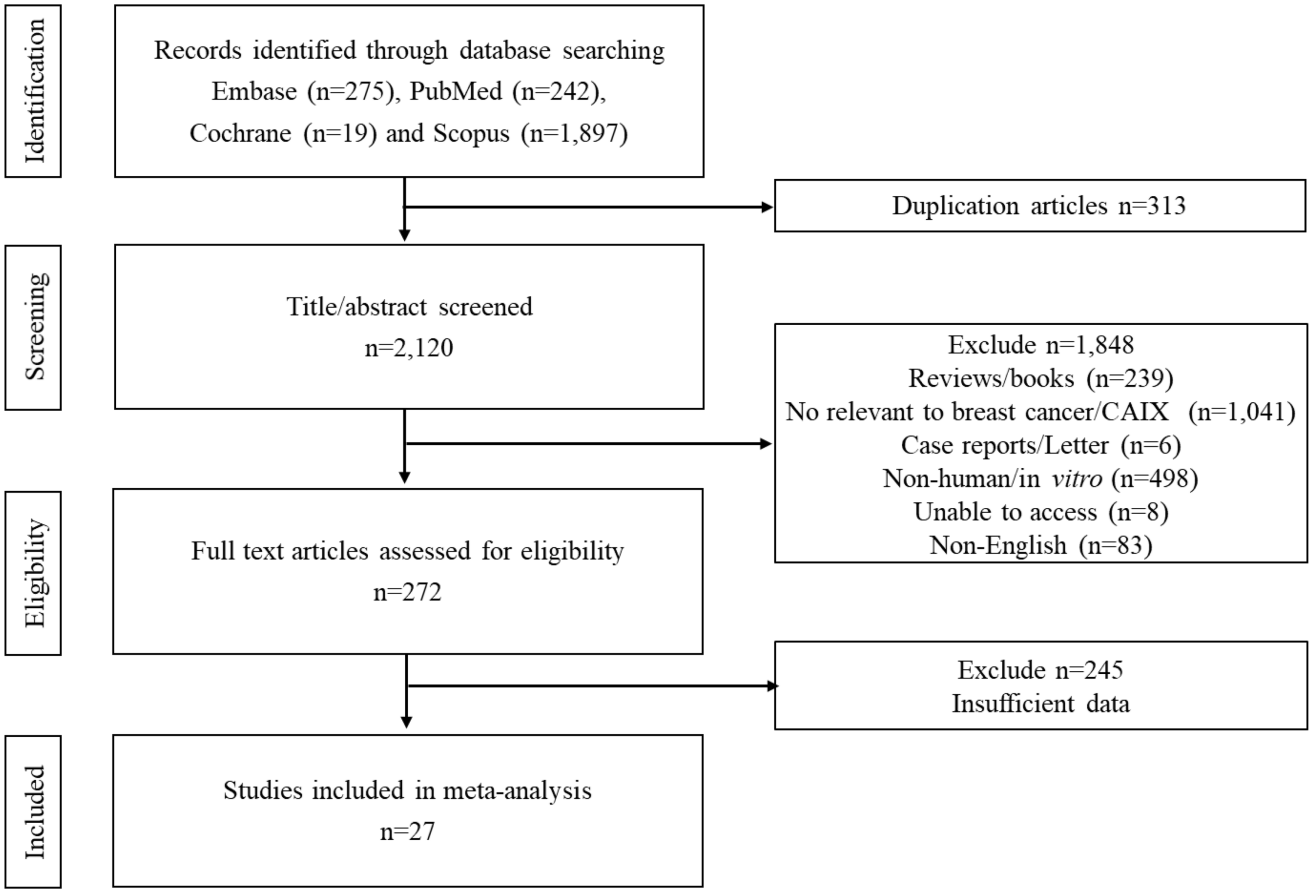
The PRISMA flow diagram for the process of study selection.

A PRISMA flow diagram for the process of study selection is summarized in Figure 1. Initially, 275 articles from EMBASE, 242 from PubMed, 19 from Cochrane, and 1,897 from Scopus were identified, and subsequently, 313 duplicated records were removed. A total of 2,120 papers were screened. A total of 1,848 studies were excluded based on the titles and abstracts resulting in 272 full texts being reviewed. Of these, 245 articles were excluded. Finally, 27 papers met the eligibility criteria (Figure 1; Table 1).

**Table 1:** Characteristics of the eligible studies for meta-analysis in this study.

References	Country	Mean age	BC subtypes (*n*)	Stage	Treatment (*n*)	IHC score method	CAIX cut-off level	CAIX high (%)	Ab clones	HR (95% CI) for DFS	*p*-value	HR (95% CI) for OS	*p*-value
Shamis et al. (26)	United Kingdom	NA	ER+ (373)	I–III	CMT (110)	Weight H score	Log-rank statistics by R Studio	9	M75	UV = 1.81 (1.12–2.92)	0.018	NA	NA
										MV = 1.04 (0.46–2.35)	0.926		
		NA	ER+ (285)	I–III	CMT (71)			28		UV = 1.64 (1.14–2.37)	0.008		
										MV = 1.74 (1.08–2.82)	0.023		
Ong et al. (22)	Singapore	55	TNBC (306)	NA	NA	I and P	≥1	39.3	NA	MV 2.77 (1.78–4.31)	<0.001	MV 2.48 (1.50–4.09)	<0.001
Li et al. (27)	China	49	ER+ (55)	Recurrence	NA	I and P	NA	34.5	ab108351	UV* 2.64 (1.28–5.44)	0.0086	NA	NA
Alves et al. (28)	Brazil	49.6	Mixed BC (196)	IIb or III	CMT (196)	I and P	≥3	7.4	ab15086	UV* 0.32 (0.19–0.55)	<0.00001	UV* 0.33 (0.15–0.66)	<0.00001
Ozretic et al. (29)	Croatia	60	TNBC (64)	NA	NA	I and P	>60	77	ab15086	NA	NA	UV 2.85 (0.36–22.25)	0.32
Jin et al. (30)	South Korea	NA	TNBC (270)	I–II	NA	NA	≥10%	21.9	NA	UV* 1.45 (0.77–2.67)	0.25	NA	NA
Chu et al. (31)	China	55.34	Mixed (149)	I–IV	CMT	I and P	Strong intensity in ≥10% cells	15	NA	MV 5.758 (2.28–14.50)	<0.001	NA	NA
Samaka et al. (32)	Egypt	48	Mixed (56)	I–IV	NA	I and P	>1%	91.1	ab107257	NA	NA	UV* 2.09 (1.05–4.19)	0.0358
Aomatsu et al. (33)	Japan	NA	Mixed (102)	IIA–IIIA	CMT (102)	I and P	Moderate to strong staining in >10% cells	46	M75	UV* 4.52 (2.05–9.97)	0.0002	UV* 3.31 (1.56–7.05)	0.0018
Deb et al. (34)	Australia	NA	Male (276)	I–IV	NA	I and P	Strong intensity in ≥10% cells	8	NA	UV 2.2 (0.8–5.7)	0.11	NA	NA
Kim et al. (35)	South Korea	52	Mixed metastasis (162)	IV	NA	I and P	≥2	19.8	NA	NA	NA	MV 1.69 (0.77–3.69)	0.189
Noh et al. (36)	South Korea	NA	ER-AR+ (127)	I–III	NA	I and P	≥2	28.7	NA	MV 2.231 (0.670–7.426)	0.191	MV 15.89 (1.82–131.6)	0.01
Betof et al. (37)	United States	48	Mixed (209)	I–III	CMT (209)	I and P	≥50	88	M75	UV* 1.75 (0.92–3.31)	0.088	UV* 2.73 (1.2–6.21)	0.0166
Kaya et al. (38)	Turkey	46	Mixed (111)	I–III	NA	I	Any staining	55.8	H-120	UV* 0.86 (0.54–1.36)	0.5253	UV* 2.77 (1.58–4.85)	0.0004
Beketic-Oreskovic et al. (39)	Croatia	61.5	Mixed (40)	I–III	NA	I and P	52.5	60	NA	UV 6.74 (2.27–20.03)	<0.001	UV 5.68 (2.11–15.31)	<0.001
										MV 4.14 (1.28–13.35)	0.018	MV 3.99 (1.38–11.59)	0.011
Lou et al. (40)	Canada	NA	Mixed (3,630)	I–III	NA	I and P	Any staining	15.6	M75	UV* 2.30 (1.91–277)	<0.00001	NA	NA
Pinheiro et al. (25)	Portugal	NA	Mixed (122)	T1-3anyN	NA	I and P	≥3	18	ab15086	UV* 2.24 (0.79–6.35)	0.1294	NA	NA
Jubb et al. (41)	United Kingdom	57 (27–80)	Mixed (151)	I–III	CMT (63)	I and P	>10%	32	M75	CAIX score 1; UV 0.63 (0.29–1.41)	0.26	NA	NA
										CAIX score 2; UV 1.24 (0.49–3.13)	0.65		
										CAIX score 3; UV 1.83 (0.86–3.89)	0.12		
Tan et al. (42)	United Kingdom	55	Mixed (407)	I–III	NA	I and P	≥10%	14	M75	UV* 1.81 (1.14–2.86)	0.0119	UV* 4.29 (2.61–7.04)	<0.00001
Crabb et al. (43)	Canada	NA	Mixed (602)	II–III	NA	NA	NA	16.7	M75	MV 1.58 (1.12–2.22)	0.008	NA	NA
Kyndi et al. (44)	Denmark	NA	Mixed (945)	II–III	NA	I and P	≥10%	16	M75	UV 1.29 (1.02–1.62)	<0.05	UV 1.3	<0.05
												(1.06–1.60)	
Hussain et al. (45)	United Kingdom	62	Mixed (144)	I–II	NA	I and P	Weak or strong staining and focal or diffuse distribution	26	M75	NA	NA	UV 2.63 (1.21–5.70)	0.01
												MV 2.43 (1.07–5.53)	0.035
Trastour et al. (46)	France	62	Mixed (132)	I–III	CMT/ET	I and P	>1%	29	M75	MV 2.0 (1.0–4.2)	0.05	NA	0.2
Brennan et al. (23)	Ireland	NA	Mixed (400)	II	ET (199)	I	Any staining	11	M75	UV* 1.62 (1.02–2.72)	0.041	UV* 1.92 (1.09–3.38)	0.0239
Generali et al. (47)	United Kingdom	NA	Mixed (166)	T2-4N0-1	CMT/ET (187)	I and P	Any staining	24.7	M75	UV* 1.79 (0.84–3.89)	0.1315	UV* 1.99 (0.79–5.02)	0.1443
Tomes et al. (48)	Canada	NA	Mixed (53)	any T,N	NA	P	NA	NA	M75	NA	NA	UV* 0.50 (0.30–0.85)	<0.0001
Chia et al. (49)	Canada	59	Mixed (103)	I–III	CMT (27)/ET (80)	I and P	≥1	48	M75	UV* 2.38 (1.34–4.22)	0.0031	UV 2.61 (1.01–6.75)	0.05

### Study characteristics

The 27 included studies were published between 2001 and 2022. DFS was reported in 22 articles, 10 of which provided HRs and 95% CIs, while the OS analysis was included in 16 articles, 7 of which provided HRs and 95% CIs (Table 1). Most of the articles (20 out of 27, 74%) were reported on mixed breast cancer subtypes and provided data on ER, PR, and/or HER2 staining, with survival analysis on all cases, regardless of the subtype. Three studies focused on TNBC, two articles on ER-positive (ER^+^), one on ER-negative (ER^−^), and one on male breast cancer. The mean age of patients was between 46 and 62 years. Fifty percent of the studies used the primary antibody clone M75 to detect the CAIX expression. In most studies (80%), the level of CAIX expression was determined by estimating both staining intensity and the percentage of tumor cells stained. The remaining studies (20%) used only intensity or percentage. The low–-high cutoff value varied across all studies. Overall, high CAIX expression in patients with breast cancer varied in each study, ranging from 8 to 91.1%. Most studies (45.5%, 12 out of 27 studies) did not report on the cellular location of CAIX expression. In 36% of studies, expression was reported in the cell membrane, in 9% of studies, CAIX expression was reported in the membrane and cytoplasm/nucleus, and in two studies, CAIX expression was reported in the exclusive cytoplasm or nuclear staining (9%).

### High CAIX was associated with poor DFS in breast cancer

**Figure 2:**
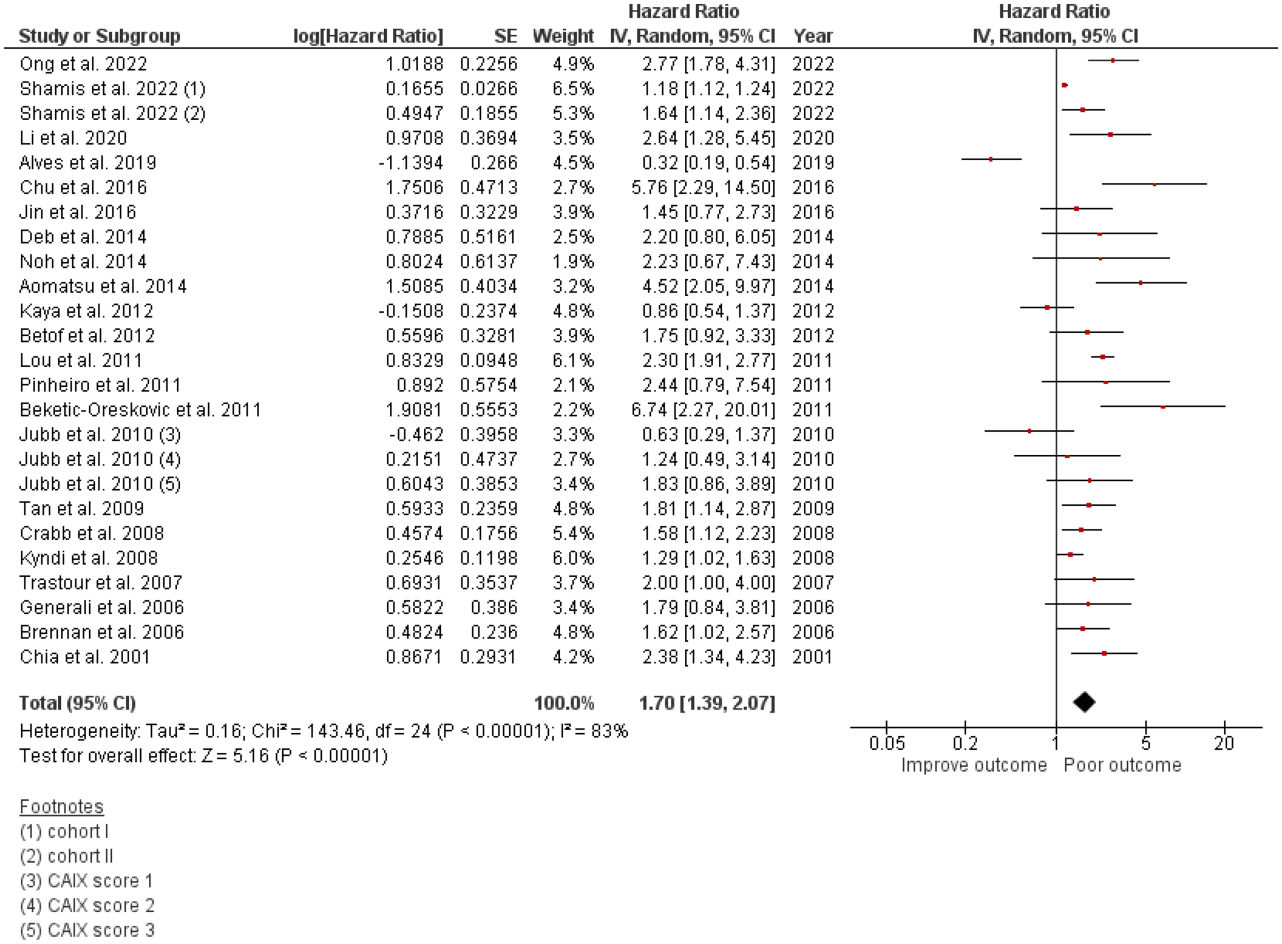
A Forest plot of HR and 95% CI for the association of CAIX with DFS of all patients with BC.

Twenty-two studies totaling 9,157 patients were analyzed for the effect of CAIX expression on DFS. Shamis et al. studied CAIX expression in two independent cohorts with specific HRs and 95% CIs and DFS in each cohort, and both cohorts were included in this meta-analysis (26). The study by Jubb et al. did not define the low/high cutoff for CAIX expression, but it provided the HR and 95% CI for each CAIX score of 1, 2, and 3 and compared each with that of the negative CAIX group (41). Hence, the HR and 95% CI for each CAIX score were included in the meta-analysis. High CAIX was significantly associated with poor DFS in patients with breast cancer (HR = 1.70, 95% CI = 1.39–2.07, *p* < 0.00001) with heterogeneity I^2^ = 83% (Figure 2).

### High CAIX was associated with poor OS in breast cancer

**Figure 3:**
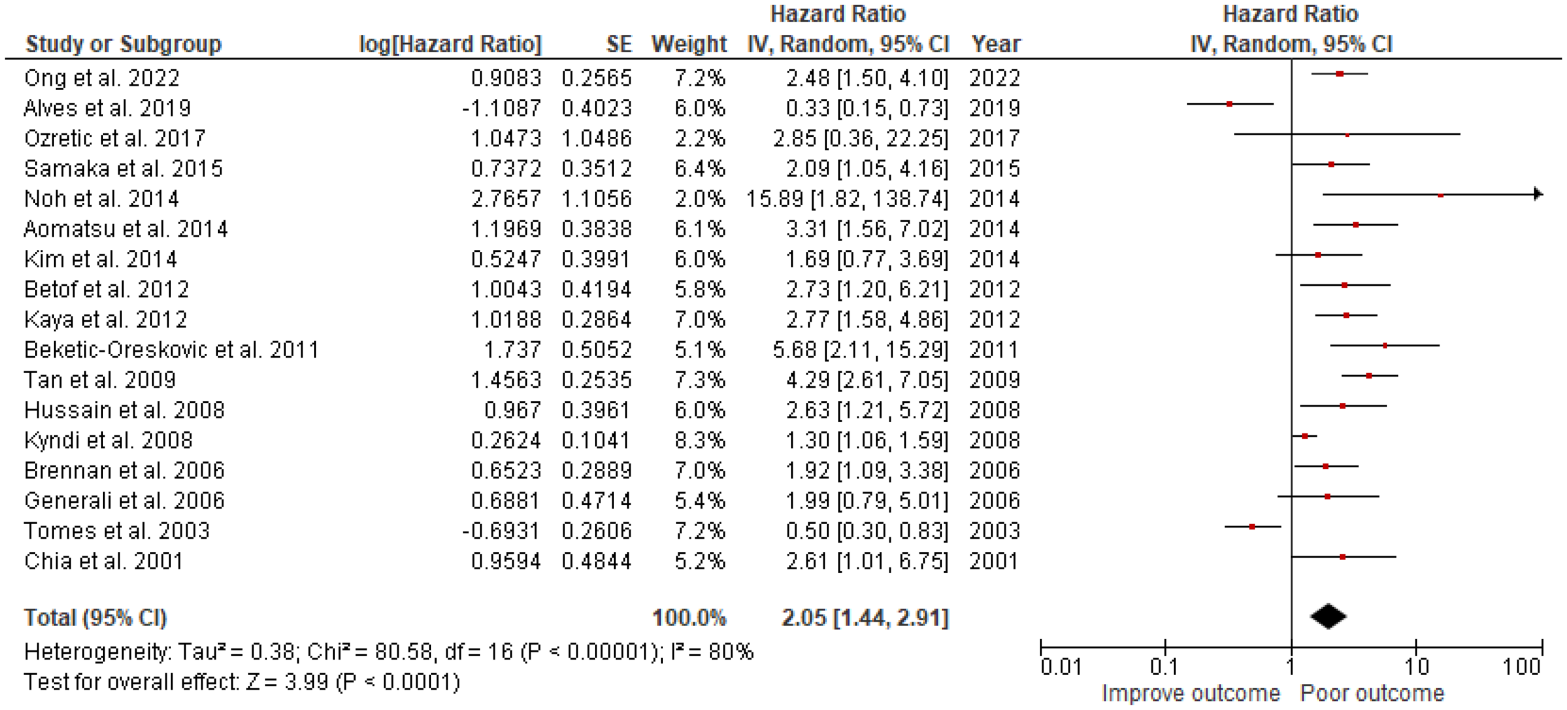
A Forest plot of HR and 95% CI for the association of CAIX with OS of all patients with BC.

A total of 3,591 patients from the selected 17 studies were investigated for the association between CAIX expression and OS. High CAIX expression was statistically significantly associated with shorter OS (HR = 2.05, 95% CI 1.44–2.91, *p* < 0.0001) with heterogeneity I^2^ = 80% (Figure 3).

### High CAIX was associated with poor OS and DFS in ER^+^ and TNBC subtypes

**Figure 4:**
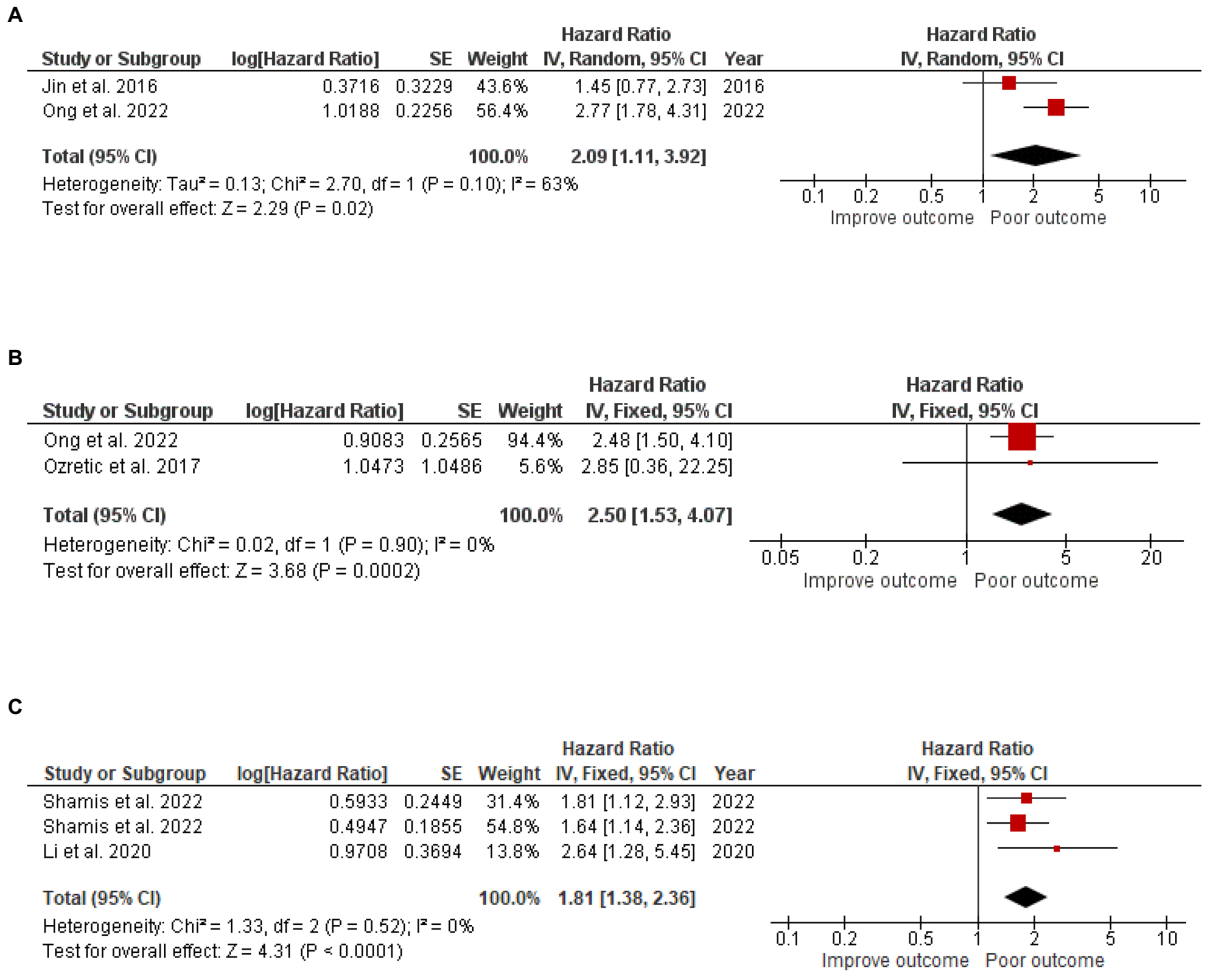
A Forest plot of HR and 95% CI for the association of CAIX with **(A)** DFS, **(B)** OS of patients with TNBC, and **(C)** DFS of patients with ER^+^ BC.

Three articles focused on the CAIX expression in 640 TNBC cases. One study reported both DFS and OS, while the other two reported either DFS or OS, resulting in 576 TNBC cases included in the DFS analysis and 370 TNBC cases included in the OS analysis. Two articles focused on CAIX expression and DFS in ER^+^ breast cancer from 731 ER^+^ breast cancer cases. The results revealed that, when compared to patients with a low CAIX expression, patients with a high CAIX expression were clearly associated with shorter DFS in TNBC (HR = 2.09, 95% CI =1.11–3.92, *p* = 0.02) with heterogeneity I^2^ = 63% and OS (HR = 2.50, 95% CI =1.53–4.07, *p* = 0.0002) without heterogeneity I^2^ = 0%; and shorter DFS in ER^+^ breast cancer (HR = 1.81 95% CI =1.38–2.36, *p* < 0.0001) without heterogeneity I^2^ = 0% (Figure 4).

### The antibody does not affect CAIX survival

**Figure 5:**
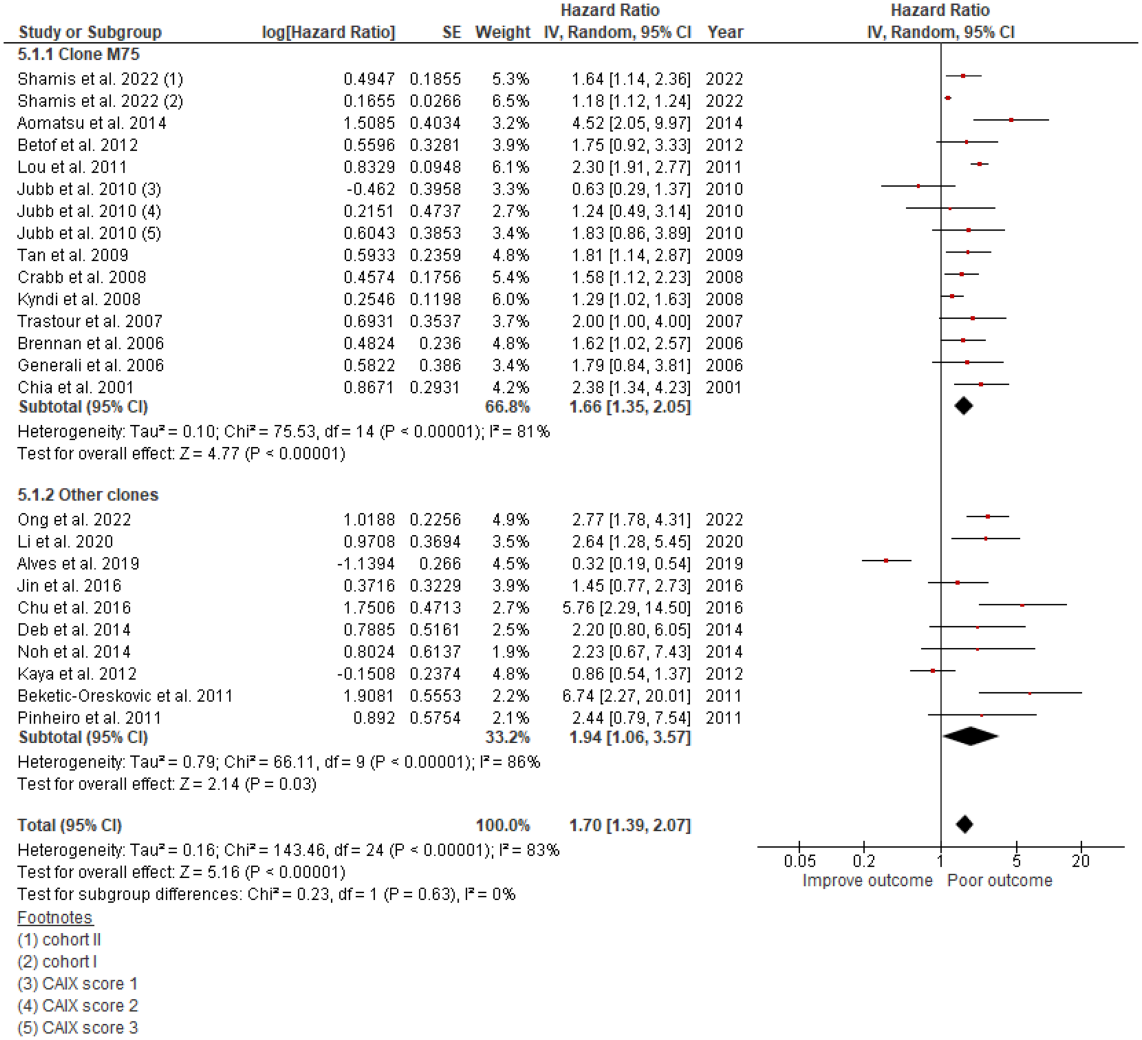
A Forest plot of HR and 95% CI for the association of CAIX expression with DFS in patients with BC stratified by the antibody clone.

**Figure 6:**
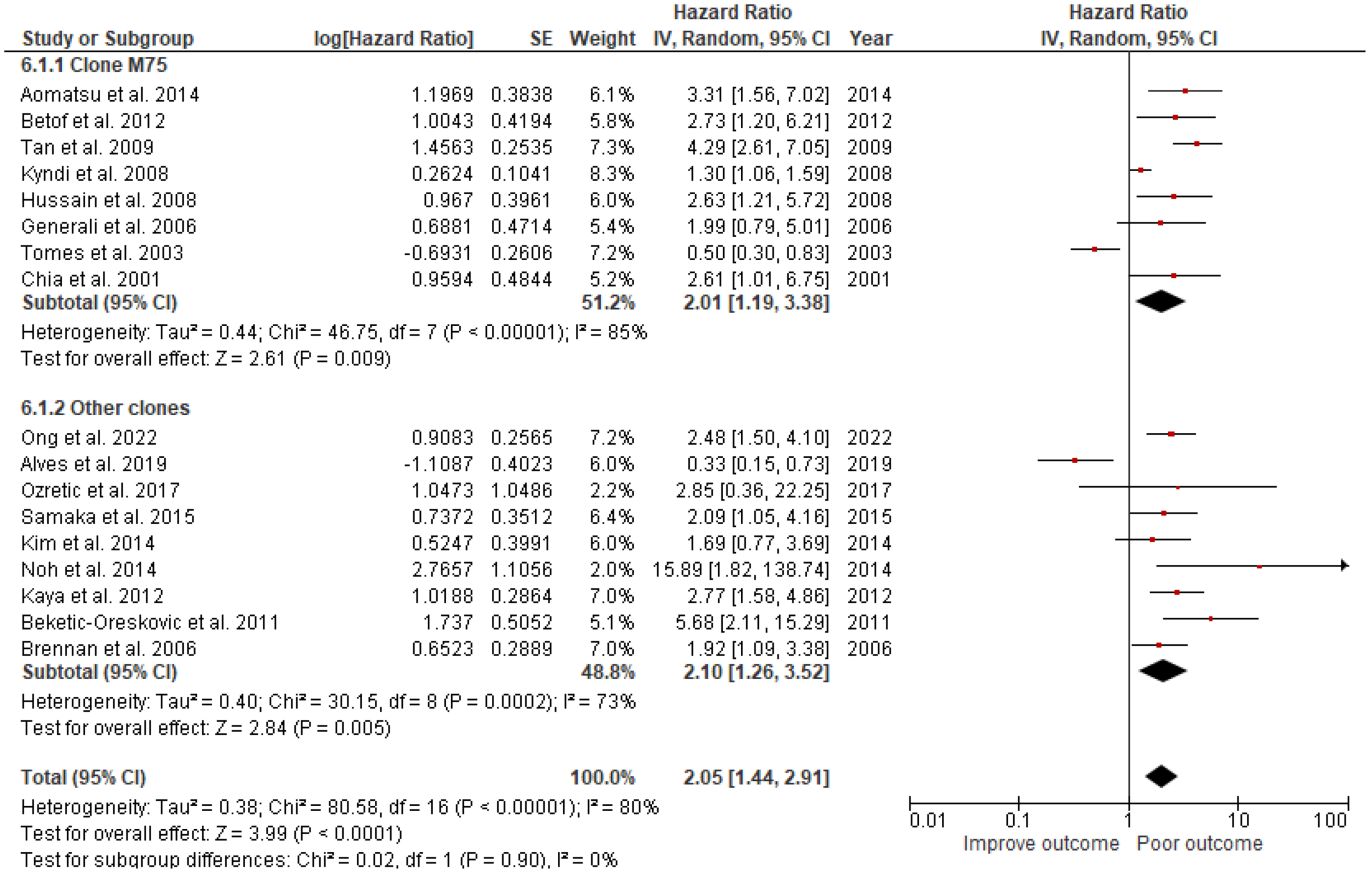
A Forest plot of HR and 95% CI for the association of CAIX expression with OS in patients with BC stratified by the antibody clone.

The studies used a variety of CAIX antibodies for IHC. Twelve studies used an M75 antibody clone: 1 from BioScience, 1 from Novus Biologicals, and 1 from Bayer, but the other 9 could not be identified. The HR for DFS was 1.66 (95% CI: 1.35–2.0, *p* < 0.00001). Clones used in other studies were as follows: 6 studies used Abcam, 1 from Cell Marque, 1 from Novus Biologicals, and 2 from Santa Cruz Biotechnology (Table 1), which also demonstrated the effect of CAIX with HR for DFS 1.94 (95% CI: 1.06–3.57; *p* < 0.0001; Figure 5). There was no significant difference between the M75 antibody and other antibodies (*p* = 0.63; Figure 5). The HR for OS in the group stained with the M75 antibody was 2.01 (95% CI: 1.19–3.38; *p* = 0.009), and it was 2.10 (95% CI: 1.26–3.52; *p* = 0.002) for the other antibody group (Figure 6). There was no significant difference between the M75 antibody and the other antibodies in terms of OS (*p* = 0.90; Figure 6).

## Discussion

This meta-analysis focused on the prognostic role of CAIX expression in breast cancer. Hypoxia, as determined by the CAIX expression, has been associated with poor survival outcomes, independent of other clinicopathological factors in many solid malignancies, including breast cancer (50). The current meta-analysis included a greater number of studies and confirmed a negative survival outcome in patients with breast cancer who had a high CAIX expression. To our knowledge, this is the first meta-analysis that has examined the CAIX expression exclusively in breast cancer. The results of this meta-analysis may lead to the use of CAIX expression as a prognostic marker, resulting in better treatment options for patients with breast cancer.

High CAIX was significantly associated with poor DFS (HR = 1.70, 95% CI = 1.39–2.07, *p* < 0.00001) and OS (HR = 2.02, 95% CI 1.40–2.91, *p* = 0.0002), despite the high heterogeneity of DFS, I^2^ = 83%, and OS, I^2^ = 81%. This heterogeneity could be explained by the bias in the scoring method and cutoff level as most of the studies determined the CAIX protein expression by the intensity and percentage of tumor cell staining and with individual cutoff levels. However, this meta-analysis did support the use of CAIX as a prognostic marker; therefore, the evaluation of CAIX expression should be considered in breast cancer.

Tumoral hypoxia has long been established as a factor in the progression and metastasis of cancer cells (51). CAIX protein expression is a reliable endogenous hypoxic marker as its expression is dependent on the HIF-1α activity (16). CAIX is a zinc metalloproteinase that is located at the transmembrane and acts to convert CO_2_ to HCO^−^_3_ and H^+^ (52). This process occurs extracellularly and results in an extracellular acidic pH. The cancer cells exploit the extracellular acidity to invade the stroma by promoting epithelial–mesenchymal transition (EMT) and cell motility as well as suppressing anti-tumor immunity by, for example, dysregulating cytotoxic T-cell functions while enhancing the function of M2 macrophages and myeloid-derived suppressor cells (MDSCs) (53, 54). These effects may explain the correlation between the increased expression of CAIX and poor survival outcomes.

Carbonic anhydrase IX is highly induced in a HIF-1-dependent manner and is constitutively expressed in VHL-defective cells. While CAXII is upregulated in VHL-defective renal tumors and induced hypoxia in tumor cells, its dependence on HIF is not well established (15). Additionally, it is well known that the tumor expression of HIF-1α and CAIX was correlated with poor patient survival, CAXII, which lacks the extracellular proteoglycan domain of CAIX implicated in cell adhesion, had a less obvious survival effect (17). CAXII expression is related to better survival statistics for patients (55–57). In breast cancer, there is a strong association between luminal cancers and CAXII expression. Moreover, CAXII is also a biomarker of favorable prognosis in lung (58) and brain (59) tumors but is associated with a poor prognosis in colorectal cancer (60).

Additionally, this meta-analysis clarified the importance of CAIX expression associated with survival outcomes in both ER^+^ and TNBC. Li et al. reported increased tamoxifen resistance in ER^+^ breast cancer with a high CAIX expression (27). Similarly, a study by Tan et al. demonstrated the adverse effect of CAIX expression on basal-like breast cancer subtypes by escalating the chemotherapy resistance (42). This may imply that CAIX overexpression is a hostile factor mediating treatment resistance. Thus, a combination of chemotherapy and CAIX inhibitors may be helpful in the prevention of chemoresistance. This meta-analysis had several limitations. The high degree of heterogeneity of the study indicated that we were unable to accurately define a CAIX expression scoring method and optimal threshold values. Further studies to standardize the IHC protocol for CAIX are needed. The publication bias might overestimate the survival outcome as articles reporting positive findings were selected.

## Conclusion

Our results highlight the importance of a high CAIX expression being associated with poor DFS and OS in patients with breast cancer. This information may be useful for future studies, leading to the incorporation of CAIX inhibitors in treatment regimens for patients with breast cancer. High-quality studies with larger homogeneous samples are required to determine the prognostic role of CAIX in different breast cancer subtypes.

## Data availability statement

The original contributions presented in the study are included in the article/supplementary material, further inquiries can be directed to the corresponding author.

## Author contributions

WN and CT contributed to the framework and the overall perspective of the study design. The literature search was carried out by WN, SY, and JP. SY and JP extracted the data and assisted with quality control. JP carried out the statistical analysis. WN wrote the manuscript and created the tables and figures. The statistical analysis was supervised and verified by JE and CT. WN, JE, and CT contributed to the study’s quality assessment and manuscript revision. JQ checked and edited the English grammar. All authors contributed to the article and approved the submitted version.

## Funding

This study received funding from the National Research Council of Thailand (NRCT) and Mahidol University (grant no. N42A650343).

## Conflict of interest

The authors declare that the research was conducted in the absence of any commercial or financial relationships that could be construed as a potential conflict of interest.

## Publisher’s note

All claims expressed in this article are solely those of the authors and do not necessarily represent those of their affiliated organizations, or those of the publisher, the editors and the reviewers. Any product that may be evaluated in this article, or claim that may be made by its manufacturer, is not guaranteed or endorsed by the publisher.
